# Effect of Voluntary Wheel-Running Exercise on the Endocrine and Inflammatory Response to Social Stress: Conditioned Rewarding Effects of Cocaine

**DOI:** 10.3390/biomedicines10102373

**Published:** 2022-09-23

**Authors:** Carmen Ferrer-Pérez, Marina D. Reguilón, José Miñarro, Marta Rodríguez-Arias

**Affiliations:** 1 Department of Psychology and Sociology, Faculty of Humanities and Social Sciences, University of Zaragoza, 44003 Teruel, Spain; 2 Department of Psychobiology, Faculty of Psychology, Universitat de València, 46010 Valencia, Spain

**Keywords:** social stress, cocaine, conditioned place preference, neuroinflammation, physical activity, voluntary wheel running

## Abstract

The present paper evaluates the effect of physical activity on the increase of the conditioned rewarding effects of cocaine induced by intermittent social stress and on the neuroinflammatory response that contributes to the enhancement of drug response. For that purpose, three studies were designed in which social stress was induced in different samples of mice through a social-defeat protocol; the mice underwent an increase of physical activity by different modalities of voluntary wheel running (continuous and intermittent access). The results showed that continuous access to running wheels prior to stress enhanced the establishment of cocaine place preference, whereas an intermittent access exerted a protective effect. Wheel running contingent to cocaine administration prevented the development of conditioned preference, and if applied during the extinction of drug memories, it exerted a dual effect depending on the stress background of the animal. Our biological analysis revealed that increased sensitivity to cocaine may be related to the fact that wheel running promotes inflammation though the increase of IL-6 and BDNF levels. Together, these results highlight that physical exercise deeply impacts the organism’s response to stress and cocaine, and these effects should be taken into consideration in the design of a physical intervention.

## 1. Introduction

From a neuroscientific perspective, drug addiction can be defined as a chronic brain disorder where there is an inability to control the consumption of a drug despite its harmful consequences [1]. In the genesis of this disorder, there is an interplay between biological and environmental factors that determine an individual’s vulnerability to initiate and progress into addiction. Among environmental variables, some are considered to be risk factors, but others can be controlled to act as a protective factor or to help in the treatment of the disorder. One of these environmental interventions that has been shown to be useful treating substance-use disorders is physical exercise [2]. Regular physical activity appears to have protective effects on the initiation of tobacco and other drugs during adolescence and early adulthood [3]. During drug-abuse cessation, physical activity has been useful helping in tobacco treatment by decreasing cravings and withdrawal symptoms, therefore reducing the relapse rate [4,5]. However, there is no empiric evidence that proves a causal effect in humans [6]. In this regard, animal models allow for a better control of contaminating variables, such as socioeconomic factors, and a better manipulation of the independent variable regulating the duration and intensity of the exercise.

Voluntary wheel running (VWR) in rodents is widely used to mimic physical aerobic training in humans [7]. In general, VWR has been highlighted as a protective environmental factor in several animal models of different psychological disorders, including drug addiction [4,8,9,10,11]. During initial contact with drugs, access to a running wheel decreases voluntary consumption of ethanol in mice [12,13]. In the same line, animals with access to an activity wheel consume less of the drug during the acquisition of the self-administration behavior of methamphetamine, cocaine, and heroin [14,15,16,17,18]. After the establishment of self-administration behavior, access to activity wheels has a protective effect by decreasing cue- and drug-induced reinstatement of cocaine administration [16] and nicotine seeking [19]. However, the results obtained from using the conditioned place preference (CPP) paradigm, which evaluates secondary motivational properties of the drug [20], are not consistent [6,14]. Some studies point to the fact that animals with access to running wheels had lower cocaine-, ethanol-, or morphine-induced CPP compared to sedentary counterparts [21,22,23,24]. However, other studies report no effects [6] or an enhancement of the rewarding potential of morphine [25] and cocaine [14], even with an increased duration of drug-associated memories [26]. Therefore, although physical activity is an appealing intervention, under certain circumstances, the literature points out that it may be ineffective or even enhance the formation of contextual drug-associated memories [27].

Stress is another environmental factor that has been deeply studied in the genesis of drug addiction. Social adverse events are one of the most important sources of stress in humans, as they are experienced more intensely than any other type of stressors [28]. Epidemiological studies show that negative social experiences, such as isolation or bullying in the workplace, are linked to higher rates of drug abuse and higher vulnerability to relapse after periods of detoxification [29,30]. Similar results have been detailed in basic research with rodent models, using the social-defeat paradigm, which is one of the most representative models in the study of psychophysiological consequences of stress [31,32]. The literature points out that intermittent experiences of social defeat induce a robust enhancement in mice sensitivity to the conditioned and unconditioned rewarding effects of cocaine [33,34]. In self-administration paradigms, defeated animals display faster acquisition and escalation of the administration behavior, are more motivated to obtain a dose of the drug, and are more vulnerable to reinstate drug seeking after a priming dose of the drug [35,36]. CPP studies have painted a similar picture, and socially stressed animals are more sensitive to small doses of cocaine, require more time to extinguish the conditioned preference, and are more susceptible to reinstate the said preference [37,38,39]. To explain this stress effect on drug response, researchers have classically focused on studying the neurobiological adaptations in the neuroendocrine axis of stress and within the reward pathway. More recently, the mediation of inflammatory processes in stress-induced vulnerability to drug addiction has also been explored [11,37,40,41]. However, the temporal pattern an intensity of social-stress experiences has to be taken into consideration, as prolonged experiences of social defeat have been reported to induce a different pattern of neuroadaptive changes [42] and even a suppression of cocaine administration in rodents (see revision in Reference [43]).

In this context, part of the utility of physical exercise as a therapeutical tool in addiction may be linked to its anti-stress and anxiolytic effects [2]. In humans, regular physical exercise has a positive effect in the management of clinical stress [44], increasing the ability to cope with daily stressful events without using drugs of abuse [45]. In preclinical research using rodents, VWR has been found to be protective against negative consequences of episodic and chronic social defeat. For instance, 21 days of VWR prior to intermittent or chronic social-defeat stress has been found to protect against the development of depression-like behaviors, such as anhedonia and social avoidance [7,46,47]. Recently, in our laboratory, we found that rodents with intermittent and controlled VWR before social-defeat experiences and maintained during the whole study were protected against the increase in ethanol intake on an oral self-administration paradigm [11]. In order to increase the understanding about which physiological mechanisms underlie the modulatory effect of physical activity over stress and drug response, in the present study, we compared the impact of four modalities of physical exercise (sedentary activity, housing with one or four voluntary running wheels, and a regimen of intermittent voluntary wheel running) in the increase of cocaine-conditioned reward induced by social stress and in the extinction of drug-associated memories. In addition, we explored different neurobiological mechanisms that could be underlying the observed effects of physical exercise. (1) As there is evidence showing that social stress can induce inflammatory states that are mediating the vulnerability to stress-related disorders [48,49] and drug addiction [37], we evaluated whether physical exercise could exert a modulatory effect over this stress-induced inflammatory response and therefore modulate drug response. (2) We also measured corticosterone levels after social-defeat stress episodes in order to determine a possible blunting effect of VWR in the neuroendocrine response of the stress. (3) Additionally, we evaluated the potential of physical exercise to increase the expression of brain-derived neurotrophic factor (BDNF) in the hippocampus and within the striatum, as these structures are key in the associative learning involved in CPP [50], and plastic changes in these areas can sustain enhanced drug learning, as reported in previous studies [9,25]. (4) Finally, we evaluated whether VWR can act as a hedonic substitute that competes with the rewarding potential of cocaine in the establishment of conditioned drug memories. The current work would increase the knowledge about the neurobiological mechanism that underlies the effect of physical exercise over the consequences of stress on drug response and drug-associated memories. This would help to optimize the designs of physical interventions, helping to choose the most beneficial regimen of exercise for the patient.

## 2. Materials and Methods

### 2.1. Animals

A total number of 238 OF1 adult mice on postnatal day (PND) 42 were acquired from Charles Rivers (Lyon, France). Upon arrival at the animal facility, a total number of 92 animals (sample for Study 1) were housed in plastic cages (59 × 38 × 20 cm), with one (1 × 4 Housing), four (4 × 4 Housing), or no voluntary running wheels (Sedentary Housing or Intermittent exercise). Another set of 96 animals (Study 2 and Study 3) were housed in groups of four in plastic cages (27 × 27 × 14 cm), without additional environmental enrichment. In addition to the experimental animals, 50 OF1 adult male mice were housed individually in order to enhance their territorial aggressiveness and were used as aggressors for the social-defeat encounters.

Regardless of the assigned experimental group, all the animals were kept in the same conditions: constant temperature; a reversed light schedule (white light on 8:00–20:00); and food and water available ad libitum, except during behavioral tests. The experimental protocol was approved by an Institutional Review Committee for the use of animal subjects (Comité d’Ética d’Experimentació i Benestar Animal, number 2015/VSC/PEA/00168). Procedures involving mice and their care were conducted according to national, regional, and local laws and regulations, which are in compliance with the Directive 2010/63/EU [51]. All efforts were made to minimize the animals’ suffering and to reduce the number of animals used. 

### 2.2. Drugs

For CPP conditioning, animals were injected intraperitoneally with a dose of 1, 3, or 10 mg/kg of cocaine hydrochloride (Alcaliber laboratory, Madrid, Spain) dissolved in physiological saline (NaCl 0.9%) and adjusted to a volume of 0.01 mL/g of weight. These doses of cocaine were selected on the basis of previous CPP studies, showing that a dose of 3 and 10 mg/kg are effective doses to induce CPP in OF1 male mice, while 1 mg/kg is considered to be a sub-threshold dose CPP in adult animals without stress or drug experiences [39,52].

### 2.3. Experimental Groups and Experimental Design 

**Figure 1:**
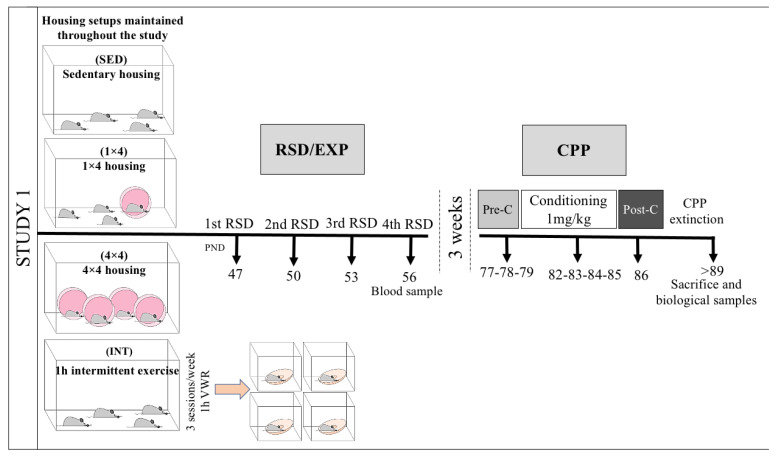
Experimental design and experimental groups of Study 1.

In Study 1 (depicted in Figure 1), the effects of being housed with continuous or intermittent access to voluntary running wheels in the long-term effects of intermittent social defeat was evaluated. Upon arrival at PND 42, animals were housed in groups of four in cages in different setups. Part of the sample was housed under standard condition, without running wheels (*n* = 48), while the remaining animals were housed in cages that contained one (1 × 4, *n* = 24) or four (4 × 4, *n* = 20) activity wheels inside their home cage; these setups were maintained until the end of the study. Within the first group of 48 animals housed without activity wheels, half of the animals were included in the sedentary condition (SED, *n* = 24), with no additional physical activity, and the other half of the animals followed an intermittent exercise regimen (INT) since the first week upon arrival and until the end the experiments (INT, *n* = 24). This regimen of intermittent exercise consisted of three sessions per week (Mondays, Wednesdays, and Fridays) of 1 h of individual access to a cage with a voluntary running wheel. Five days after the arrival to the animal facility, on PND 47, half of the mice in each experimental condition underwent a repeated social defeat (RSD) stress protocol (SED–RSD *n* = 12; 1 × 4-RSD, *n* = 12; 4 × 4-RSD, *n* = 10; INT–RSD, *n* = 12), while the other half underwent the exploration protocol (EXP), which involves a similar manipulation but without experiencing social-defeat stress (SED–EXP, *n* = 12; 1 × 4-EXP, *n* = 12; 4 × 4-EXP, *n* = 10; INT–EXP, *n* = 12). On PND 56, 30 min after the last episode of social defeat or EXP, blood samples were taken in order to evaluate corticosterone levels. Subsequently, 21 days after, the CPP procedure induced by 1 mg/kg was initiated (from PND 77 to 86). Biological samples were taken 24 h after confirmation of the extinction of cocaine-conditioned place preference.

**Figure 2:**
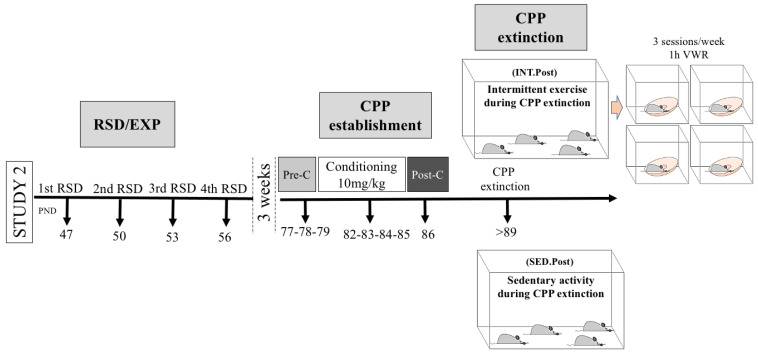
Experimental design and experimental groups of Study 2.

In Study 2 (depicted in Figure 2), we followed a similar procedure. Animals arrived on PND 42 at the animal facility and were housed under standard condition, without activity wheels. On PND 47, part of this second set of animals underwent the RSD protocol, while the remaining mice were included under the stress control condition (EXP). Three weeks after the last episode of social stress or control, animals underwent a place preference conditioning protocol with an effective dose of 10 mg/kg cocaine (from PND 77 to 86). After CPP establishment, and until the end of the study, half of the animals initiated a regimen of intermittent exercise during extinction of drug-associated memories identical to the one previously explained (INT.Post). Animals in this activity condition (EXP–INT.Post, *n* = 9; RSD–INT.Post, *n* = 11) underwent three sessions per week of 1 h of individual access to a cage with a voluntary running wheel. The remaining animals did not increase their physical activity as they continued with their sedentary routine until the end of the study (EXP–SED.Post, *n* = 9; RSD–SED.Post, *n* = 13). The extinction of the conditioned preference was evaluated in all animals two times per week (Tuesdays and Thursdays) with free drug extinction sessions. The design of Study 2 allowed us to evaluate if an enhancement of physical activity can modulate the extinction of drug-associated memories.

**Figure 3:**
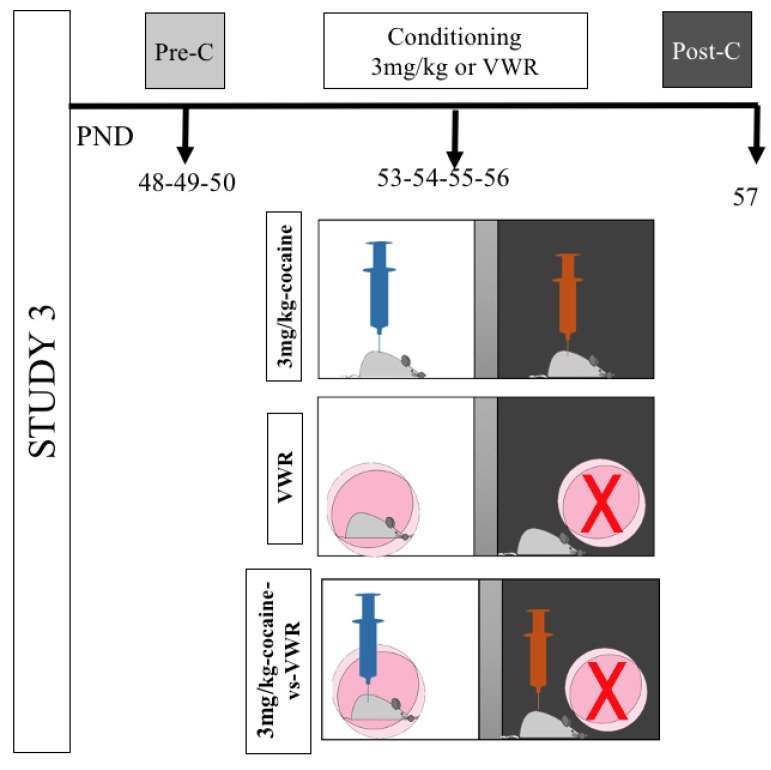
Experimental design and experimental groups of Study 3. In the diagram, blue syringes represent the injection of saline while orange syringes represent the injection of 3 mg/kg cocaine. Active running wheels are depicted as a pink circle while blocked wheels (inactive) are represented as a pink circle with a red “X”.

In Study 3 (depicted in Figure 3), the rewarding potential of VWR to establish a CPP and to compete against cocaine reinforcement was analyzed. One group of animals (VWR group, *n* = 21) underwent a CPP protocol in which an active running wheel was paired to one compartment and the opposite compartment was paired with a blocked wheel (inactive) that did not allow for running. A second group of animals (3 mg/kg cocaine, *n* = 15) underwent a traditional CPP protocol with the pairing of a dose of 3 mg/kg cocaine (pictured in orange) with one compartment and an injection of saline (pictured in blue) in the other (3 mg/kg cocaine, *n* = 15). Finally, in the third group (3 mg/kg cocaine vs. VWR, *n* = 17), an active running wheel and a saline injection were paired in one compartment, while a dose of 3 mg/kg cocaine and an inactive wheel were paired in the other compartment.

### 2.4. Apparatus and Procedures

#### 2.4.1. Voluntary Running Wheels

Inside the home cages and for CPP induction, we employed traditionally mounted vertical running wheels (15 cm diameter) with a solid surface. For intermittent exercise condition, low-profile running wheels (Med Associates Inc., Fairfax, VT, USA) were employed.

#### 2.4.2. Procedure of Repeated Social Defeat (RSD)

The social-defeat protocol was carried out by following the procedure previously validated and described in detail by Ferrer-Perez and coworkers [33,37,38]. Briefly, each experimental animal under the RSD condition experienced 4 intermittent episodes of social defeat. Each episode began by introducing the “intruder” experimental animal into the home cage of the aggressive “resident” (the aggressive opponent) protected by a wire-mesh wall for 10 min. During this initial phase, the experimental animal was protected from direct attack but exposed to social interaction and species-typical threats from the male aggressive resident. Then the wire mesh was removed from the cage to allow confrontation between the two animals for a 5 min period. In the third phase, the wire mesh was returned to the cage to separate the two animals once again for another 10 min. The criterion used to define an animal as defeated was the adoption of a specific posture signifying defeat, characterized by an upright submissive position, limp forepaws, upwardly angled head, and retracted ears [53]. In order to minimize the physical wounding during social defeats, the 5-min direct encounters were interrupted if the intruder displayed a submissive supine posture for more than 8 s or if it was bitten by the aggressor more than 12 times [54].

#### 2.4.3. Conditioned Place Preference (CPP)

Place conditioning consisted of three phases and took place during the dark cycle, following an unbiased procedure in initial spontaneous preference terms. For place conditioning, sixteen identical Plexiglas boxes with black and white equally sized compartments (30.7 × 31.5 × 34.5 cm) separated by a gray central area (13.8 × 31.5 × 34.5 cm) were used. In brief, during preconditioning (Pre-C), the time spent by the animal in each compartment over a 15-min period was recorded. Mice showing a strong unconditioned aversion (less than 33% of the time spent in both compartments) or a preference (more than 67%) for any compartment were excluded from the study.

In the second phase (conditioning), animals underwent two pairings per day: subjects received an injection of physiological saline before being confined to the vehicle-paired compartment for 30 min and, after an interval of 4 h, received cocaine immediately before being confined to the drug-paired compartment for 30 min. All animals in Study 1 were conditioned with a dose of 1 mg/kg of cocaine, while the animals in Study 2 and a group of 15 animals from Study 3 followed the same protocol, but with a higher dose of cocaine (10 or 3 mg/kg).

Additionally, in Study 3, two experimental groups underwent a variation of this procedure. Following a similar protocol, one group (VWR, *n* = 21) was confined to one compartment with a free running wheel for 30 min and, after an interval of 4 h, the group was confined to the other compartment with a locked wheel. The remaining group of animals (3 mg/kg cocaine vs. VWR, *n* = 17) experienced a conditioning procedure with the pairing of an active wheel and an injection of saline in one compartment and a dose of 3 mg/kg cocaine and a locked wheel in the other compartment. 

In the third phase (post-conditioning; Post-C), the time spent by mice in each compartment during the 15 min observation period was recorded. The difference in seconds between the time spent in the drug-paired or wheel-paired compartment in the Post-C test and that spent in the Pre-C test is a measure of the degree of conditioning induced. If this difference is positive, then the drug administration or the activity in the wheel is considered to have induced a preference for the paired compartment, whereas the opposite indicates the development of aversion. 

All groups from Study 1 and Study 2 for which a preference for the drug-paired compartment was established underwent two extinction sessions per week (Tuesdays and Thursdays), which consisted of placing the mice in the apparatus for 15 min without the administration of cocaine. This was repeated until the time spent in the drug-paired compartment by each group was similar to that of the Pre-C. Then the effects of non-contingent administration of a priming dose of cocaine were evaluated 24 h after the confirmation of extinction. Reinstatement tests were the same as those for the Post-C (free ambulation for 15 min), except for the fact that mice were tested 15 min after administration of a priming dose (half of the dose used for conditioning). This procedure was repeated with progressively lower priming doses until a non-effective priming injection was determined.

#### 2.4.4. Collection and Analysis of Biological Samples

Blood sampling for corticosterone determination was performed by following the same procedure previously published in Montagud-Romero and coworkers [55] and collected 30 min after the fourth episode of social defeat. Using the tail-nick procedure, the animal was immobilized and wrapped in a cloth so as to decrease experimental stress, and through a 2 mm incision at the end of the tail artery, 50 μL of blood was collected. Blood samples were kept on ice, and plasma was separated from whole blood by centrifugation (5 min, 5000 G), transferred to sterile microcentrifuge tubes, and stored at −80 °C until corticosterone determination.

Brain-tissue sampling and plasma for IL-6 and BDNF determination was carried out 24 h after finishing the CPP procedure in Study 1. Animals were sacrificed by cervical dislocation and then decapitated to collect blood from the neck in tubes coated with heparin. Blood samples were kept on ice, and plasma was separated from whole blood by centrifugation (5 min, 5000 G) and transferred to sterile 0.2 mL microcentrifuge tubes. To obtain striatal and hippocampal tissue samples, brains were removed immediately after decapitation and dissected, following the procedure described by Heffner and coworkers [56]. Plasma and tissue samples were stored at −80 °C until IL-6 and BDNF determinations. 

For the determination of plasmatic corticosterone levels, samples were diluted with the reagents provided in the ELISA kit obtained from Enzo Life (Ref: ADI-900-097) and analyzed by following the manufacturer’s instructions. For brain BDNF and IL-6 determinations, tissues were first homogenized and prepared by following the procedure previously described in detail in Ferrer-Perez and coworkers [37]. For striatal, hippocampal, and plasmatic IL-6 concentration, we used a Mouse IL-6 ELISA Kit obtained from Abcam (Ref: ab100712), and for BDNF quantification, we used the Emax ImmunoAssay System obtained from Promega (Ref: G7610), and they were used according to the manufacturer’s instructions. Final tissue concentrations were normalized to each sample’s protein levels determined by the Bradford assay from ThermoFisher (Ref: 23227).

ELISA test results were read by using an iMark microplate reader (Bio-RAD, Hercules, CA, USA) controlled by Microplate Manager 6.2 software (Bio-Rad Laboratories, Hercules, CA, USA), and the final results were expressed in pg/mg for brain tissue samples and in pg/mL for plasma.

### 2.5. Statistical Analyses 

In Study 1, the establishment of CPP was determined by using a two-way ANOVA with two between-subjects variables, namely stress, with two levels (RSD and EXP), and activity, with four levels (SED, 1 × 4, 4 × 4, and INT); and a within-subjects variable, days, with two levels (Pre-C and Post-C). Additionally, one-way ANOVA with a within-subjects variable—days, with two levels (Pre-C and Post-C)—was carried out for the data of each activity condition (SED, 1 × 4, 4 × 4, and INT). For the analysis of the biochemical data, a two-way ANOVA with the same two between-subjects variables (stress and activity) was considered to analyze the data of corticosterone IL-6 and BDNF levels. 

Similarly, the establishment of CPP in Study 2 was determined by using a two-way ANOVA with two between-subjects variable, namely stress, with two levels (RSD and EXP), and exercise during extinction, with two levels (SED.Post and INT.Post). Extinction and reinstatement values were analyzed by Student’s *t*-test, and the time required for the preference to be extinguished in each animal was analyzed by means of the Kaplan–Meier test with Breslow (generalized Wilcoxon) comparisons [57]. Although the mean of the group as a whole determined the day on which extinction was considered to have been achieved, preference was considered to be extinguished when a mouse spent 378 s or less in the drug-paired compartment on two consecutive days. We chose this time based on the values of all the Pre-C tests performed in the study (mean =368 s). When the preference was not extinguished in an animal, it was assigned the number of days required for extinction for the group as a whole.

To analyze the CPP in Study 3, the Pre-C and Post-C data were analyzed with a two-way ANOVA with one between-subjects variable, namely CPP modality, with three levels (VWR, 3 mg/kg cocaine, and 3 mg/kg cocaine vs. VWR); and a within-subjects variable—days, with two levels (Pre-C and Post-C)—was employed. 

Data are presented as mean ± SEM, and a *p*-value < 0.05 was considered statistically significant. Analyses were performed by using SPSS v26 (IBM, Armonk, NY, USA). In all cases, post hoc comparisons were performed with Bonferroni tests.

## 3. Results

### 3.1. Physical Activity Modulates the Increase in Plasmatic Corticosterone Levels after an Episode of Social Defeat

**Figure 4:**
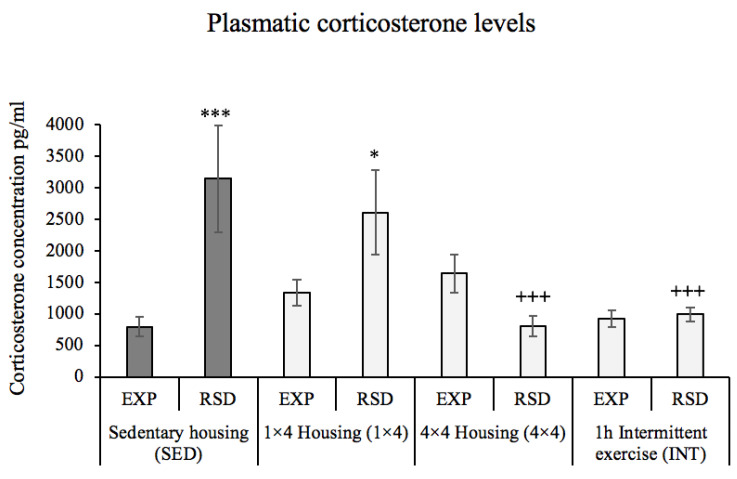
Physical activity in the form of voluntary wheel running modulates the increase in plasmatic corticosterone levels after an episode of social defeat. Mice in Study 1 were randomly assigned to the following groups, according to physical activity condition: sedentary housing (SED–EXP *n* = 9; SED–RSD *n* = 9); one activity wheel per cage (1 × 4-EXP *n* = 8; 1 × 4-RSD *n* = 8); four activity wheels per cage (4 × 4-EXP *n* = 8; 4 × 4-RSD *n* = 8); regimen of intermittent exercise (INT–EXP *n* = 7; INT–RSD *n* = 8). Data are presented as mean values of corticosterone (pg/mL in plasma) ± SEM; * *p* < 0.05; *** *p* < 0.001 significant difference in corticosterone levels with respect the corresponding EXP group; +++ *p* < 0.001 significant difference in corticosterone levels vs. SED–RSD.

The data of the corticosterone concentration after the fourth social-defeat episode in Study 1 are depicted in Figure 4. The ANOVA showed a significant effect of the variables activity [F(3,57) = 3.629, *p* = 0.018] and stress [F(1,57) = 6.899, *p* = 0.011] and the interaction activity × stress [F(3,57) = 6.806, *p* = 0.001]. The post hoc analysis showed that defeated mice housed in the sedentary housing condition (SED–RSD) or with one activity wheel (1 × 4-RSD) had higher corticosterone levels 30 min after the fourth episode of social defeat when compared to the levels obtained in non-stressed animals under the same housing condition (SED–EXP, *p* = 0.001; and 1 × 4-EXP, *p* = 0.024). The analysis also showed that, among the stressed animals, significant lower levels of corticosterone were recorded in the groups of animals housed with four activity wheels (4 × 4-RSD, *p* = 0.001) and with a regimen of intermittent exercise (INT–RSD, *p* = 0.001) when compared to RSD animals under sedentary housing condition (SED–RSD). No differences were obtained in the corticosterone levels of EXP animals between housing conditions. 

### 3.2. The Increase of Physical Activity Enhanced Cocaine-Conditioned Rewarding Effects

The ANOVA performed with CPP data showed a significant effect of the variable days [F(1,84) = 64.5, *p* < 0.001] and an effect of the interaction of the variables days × activity [F(3,84) = 8.679, *p* < 0.001], as mice housed with one (1 × 4) or four activity wheels (4 × 4) significantly increased the time spent on drug-paired compartment during Post-C test in comparison with sedentary animals (SED) or in animals on a regimen of intermittent exercise (INT) (*p* < 0.05 for 1 × 4, and *p* < 0.001 for 4 × 4). 

**Figure 5:**
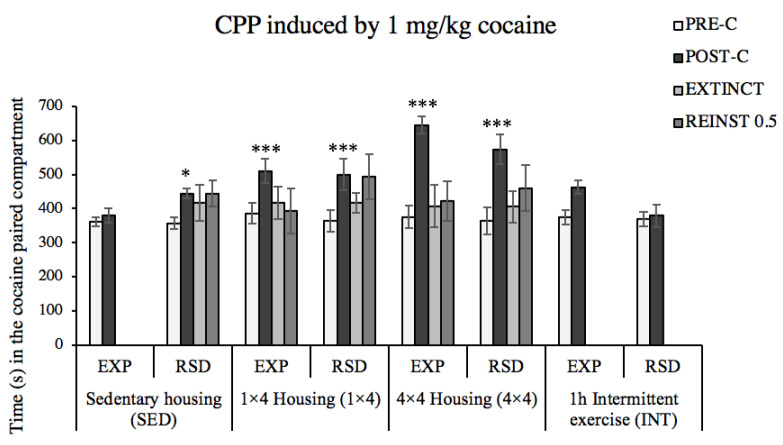
The increase of physical-activity-enhanced cocaine-conditioned rewarding effects. Animals were randomly assigned to the following groups according to their physical activity condition: sedentary housing (SED–EXP *n* = 12; SED–RSD *n* = 12); one activity wheel per cage (1 × 4-EXP *n* = 12; 1 × 4-RSD *n* = 12); four activity wheels per cage (4 × 4-EXP *n* = 10; 4 × 4-RSD *n* = 10); regimen of intermittent exercise (INT–EXP *n* = 12; INT–RSD *n* = 12). The bars represent the time (s) spent in the drug-paired compartment before conditioning sessions in the Pre-C test (white bars), and after conditioning sessions in the Post-C test (black bars), in the last extinction session (EXTINCT, light gray bars), and in the reinstatement (REINST) test (dark gray bars). Data are presented as mean values ± SEM. Bonferroni’s test * *p* < 0.05; *** *p* < 0.001 significant difference in the time spent in the drug-paired compartment vs. corresponding Pre-C.

Additionally, ANOVAs for each physical activity condition were performed (Figure 5). The ANOVA for the CPP data of sedentary (SED) mice showed an effect of the variable days [F(1,22) = 18.42, *p* < 0.001] and the interactions of days × activity [F(1,22) = 7.772, *p* = 0.011]. The post hoc analysis showed that only socially defeated animals significantly increased the time spent in the drug-paired compartment in the Post-C test (*p* < 0.001). Data from animals housed with activity wheels showed an effect of the variable days in both modalities 1 × 4 [F(1,22) = 13.145, *p* = 0.001] and 4 × 4 [F(1,18) = 35.122, *p* < 0.001], as stressed and non-stressed animals in these activity conditions developed CPP. Finally, no significant changes in CPP were observed in animals on a regimen of intermittent exercise (INT). No differences were observed in the time needed to extinguish these preferences (two sessions), and neither of the groups showed reinstatement after a priming dose with 0.5 mg/kg of cocaine.

### 3.3. Physical Activity in the Form of Wheel Running Increases IL-6 Concentration in Plasma, the Striatum, and the Hippocampus

**Figure 6:**
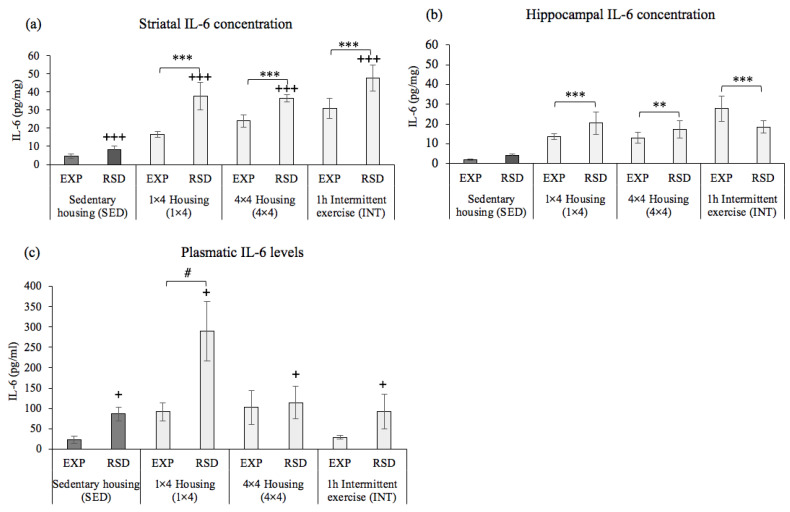
(**a**) Effects of voluntary wheel running on striatal levels of IL-6, (**b**) Effects of voluntary wheel running on hippocampal levels of IL-6, (**c**) Effects of voluntary wheel running on plasmatic levels of IL-6. Sedentary housing (SED–EXP *n* = 6; SED–RSD *n* = 6); one activity wheel per cage (1 × 4-EXP *n* = 8; 1 × 4-RSD *n* = 8); four activity wheels per cage (4 × 4-EXP *n* = 8; 4 × 4-RSD *n* = 7); regimen of intermittent exercise (INT–EXP *n* = 7; INT–RSD *n* = 7). Data are presented as mean values of IL-6 (pg/mg protein in brain tissue and pg/mL in plasma) ± SEM. Bonferroni’s test ** *p* < 0.01; *** *p* < 0.001 significant difference in IL-6 levels vs. sedentary housing (SED); + *p* < 0.05; +++ *p* < 0.001 significant difference in IL-6 levels between EXP vs. RSD; # *p* < 0.05 significant difference in IL-6 levels between 1 × 4 vs. SED and INT.

ANOVAs of IL-6 levels in the striatum, the hippocampus, and in plasma are represented in Figure 6. The results of IL-6 striatal levels (Figure 6a) showed a significant effect of the variable activity [F(3,48 = 17.988, *p* = 0.001] and stress [F(1,54) = 18.519, *p* = 0.001]. The post hoc analysis showed that animals under sedentary housing condition (SED) had significantly lower levels of striatal IL-6 compared to the animals that were exposed to VWR (*p* = 0.001 in all cases). On the other hand, the striatal IL-6 levels among socially stressed animals (SED–RSD, 1 × 4-RSD, 4 × 4-RSD, and INT–RSD) were higher (*p* = 0.001) compared to those of their corresponding non-stressed groups (SED–EXP, 1 × 4-EXP, 4 × 4-EXP, and INT–EXP).

The results of IL-6 levels in the hippocampus (Figure 6b) showed a significant effect of the variable activity [F(3,48) = 9.606, *p* = 0.001]. The post hoc analysis showed that animals under sedentary housing conditions (SED) had significantly lower levels of striatal IL-6 compared to animals housed with one (1 × 4, *p* = 0.001) or four (4 × 4, *p* = 0.005) activity wheels, or those in intermittent exercise (INT, *p* = 0.001).

Finally, the ANOVA performed with IL-6 levels in plasma (Figure 6c) showed a significant effect of the variables activity [F(3,48) = 4.258, *p* = 0.01] and stress [F(1,48) = 4.158, *p* = 0.047]. The post hoc analysis showed that animals housed with one activity wheel (1 × 4) had significantly higher levels of plasmatic IL-6 compared to animals under sedentary housing (SED, *p* = 0.025) and under a regimen of intermittent activity (INT, *p* = 0.022). As obtained in the striatum, the plasmatic IL-6 levels among socially stressed animals (SED–RSD, 1 × 4-RSD, 4 × 4-RSD, and INT–RSD) were higher (*p* < 0.047) compared to those of non-stressed animals (SED–EXP, 1 × 4-EXP, 4 × 4-EXP, and INT–EXP).

### 3.4. Physical Activity in the Form of Wheel Running Increases BDNF Concentration in the Striatum and the Hippocampus

**Figure 7:**
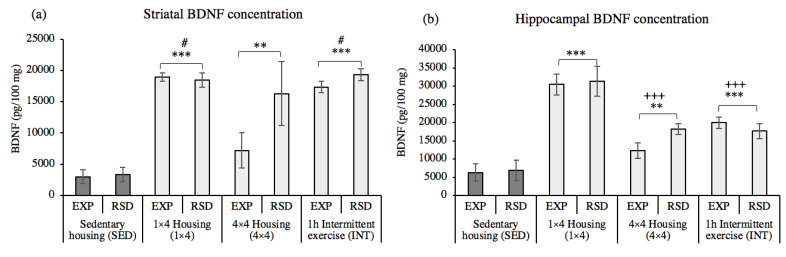
(**a**) Effects of voluntary wheel running on striatal BDNF levels, (**b**) Effects of voluntary wheel running on hippocampal BDNF levels. Sedentary housing (SED–EXP *n* = 5; SED–RSD *n* = 7); one activity wheel per cage (1 × 4-EXP *n* = 7; 1 × 4-RSD *n* = 6); four activity wheels per cage (4 × 4-EXP *n* = 9; 4 × 4-RSD *n* = 6); regimen of intermittent exercise (INT–EXP *n* = 7; INT–RSD *n* = 7). Data are presented as mean values of BDNF (pg/100 mg protein) ± SEM; ** *p* < 0.01; *** *p* < 0.001 significant difference in BDNF levels vs. sedentary housing (SED); # *p* < 0.05 significant difference in BDNF levels vs. 4 × 4; +++ *p* < 0.001 significant difference in BDNF levels vs. 1 × 4.

The ANOVA performed for BDNF levels in the striatum (Figure 7a) showed an effect of the variable activity [F(3,46) = 19.302, *p* = 0.001]. Animals housed with one (1 × 4, *p* = 0.001) or four (4 × 4, *p* = 0.003) activity wheels, or with a regimen of intermittent activity (INT, *p* = 0.001) had higher striatal BDNF levels than animals with sedentary activity (SED). Additionally, BDNF levels in animals housed with four activity wheels (4 × 4) were lower when compared to animals housed with one activity wheel (1 × 4, *p* = 0.019) or with intermittent access (INT, *p* = 0.025).

The ANOVA performed for the BDNF levels in the hippocampus (Figure 7b) showed an effect of the variable activity [F(3,46) = 29.513, *p* = 0.001]. Similar to that obtained in the striatum, animals housed with one (1 × 4, *p* = 0.001) or four (4 × 4, *p* = 0.009) activity wheels, or with a regimen of intermittent exercise (INT, *p* = 0.001), had higher BDNF levels in the hippocampus than animals with sedentary physical activity (SED). Additionally, these BDNF levels were higher in animals housed with one activity wheel (1 × 4) when compared with the levels of animals housed with four activity wheels (4 × 4, *p* = 0.001) or with intermittent access (INT, *p* = 0.001).

### 3.5. A Dose of 10 mg/kg of Cocaine Induced CPP and an Intermittent Regimen of Physical Exercise (VWR) Modulates Its Extinction

**Figure 8:**
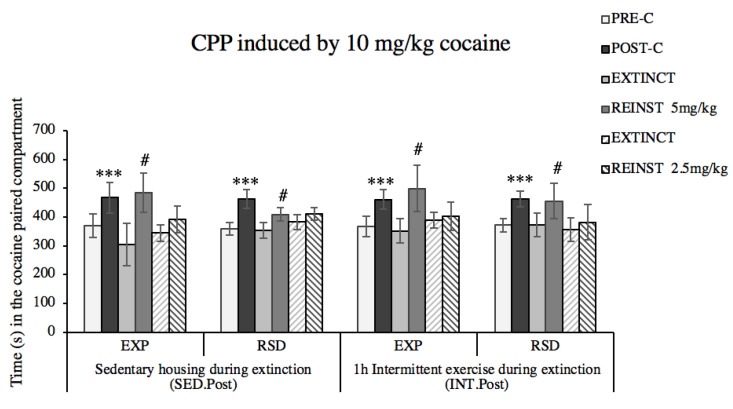
A dose of 10 mg/kg of cocaine induced CPP in all experimental groups. Animals were randomly assigned to the following groups according to their social-stress history and the physical-activity condition during extinction of drug-associated memories: non-stressed animals with sedentary activity during extinction (EXP–SED.Post) or with a regiment of intermittent exercise (EXP–INT.Post); socially defeated animals with sedentary activity during extinction (RSD–SED.Post) or with a regiment of intermittent exercise (RSD–INT.Post). The bars represent the time (s) spent in the drug-paired compartment before conditioning sessions in the PRE-C test (white bars), and after conditioning sessions in the POST-C test (black bars), in the first extinction sessions (EXTINCT, light gray bars), and in the reinstatement with 5 mg/kg cocaine (REINST 5 mg/kg cocaine, dark gray bars), in the second extinction sessions (EXTINCT, light gray striped bars), and in the reinstatement with 2.5 mg/kg cocaine (REINST 2.5 mg/kg cocaine, dark gray striped bars). Data are presented as mean values ± SEM. Bonferroni’s test # *p* < 0.05; *** *p* < 0.001 significant difference in the time spent in the drug-paired compartment vs. corresponding Pre-C.

The ANOVA of the CPP data (Figure 8) showed a significant effect of the variable days [F(1,30) = 27.028, *p* < 0.001], as all groups spent significantly more time in drug-paired compartment during Post-C compared to Pre-C. 

**Figure 9:**
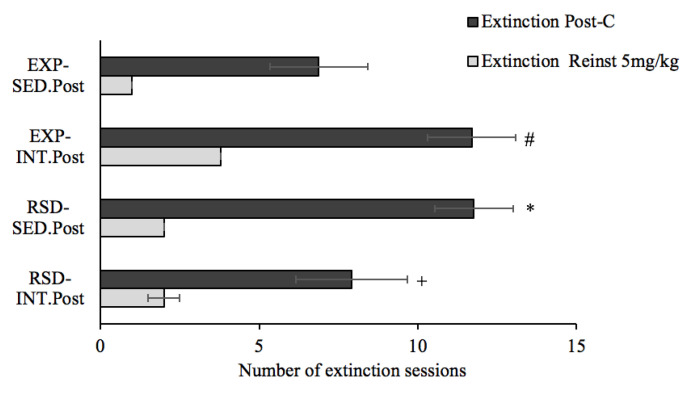
An increase of physical exercise during the extinction of drug-associated memories modifies the number of sessions needed to extinguish CPP induced by 10 mg/kg of cocaine. Black bars represent the mean of sessions required to extinguish the preference induced by 10 mg/kg cocaine, while gray bars represent the sessions required to extinguish the reinstated preference with a priming dose of 5 mg/kg cocaine; * *p* < 0.05 significant difference with respect EXP–SED.post group; # *p* < 0.05 significant difference with respect EXP–SED.post; + *p* < 0.05 significant difference with respect RSD–SED.post.

All groups were subjected to extinction sessions, and the Kaplan–Meier analysis (Figure 9) revealed that, within animals housed under standard activity condition (SED), more time was required to achieve extinction in those with previous social-defeat experiences (RSD–SED.post) than in non-stressed animals (EXP–SED.post) (χ^2^ = 4.035, *p* = 0.045). Kaplan–Meier analysis also revealed that the increase of physical activity through a regiment of intermittent exercise (INT.post) modulate the extinction of drug-associated memories. Non-stressed animals in the condition of intermittent exercise (EXP–INT.post) required more extinction sessions than sedentary counterparts (EXP–SED.post) (χ^2^ = 4.373, *p* = 0.037). However, extinction was achieved earlier in physically active socially defeated animals (RSD–INT.post) when compared to their sedentary counterparts (RSD–SED.post) (χ^2^ = 4.307, *p* = 0.038). For non-stressed mice, a negative hazard ratio of 0.42 (95% CI 0.16–1.08) was found, as VWR decreased in a 68% the probability that mice extinguish drug-induced place preference. On the other hand, in socially defeated mice, VWR exhibited an opposite effect with a positive hazard ratio of 2.25 (95% CI 0.53–9.046) favoring the probability of extinction in a 125%. Reinstatement of the preference was obtained in all the groups after a priming dose of 5 mg/kg of cocaine. No differences were observed in the time needed to extinguish this preference, and neither of these two groups showed reinstatement after priming with 2.5 mg/kg cocaine.

### 3.6. Voluntary Wheel Running Induced CPP and Neutralized the Development of Preference for an Effective Dose of Cocaine (3 mg/kg)

**Figure 10:**
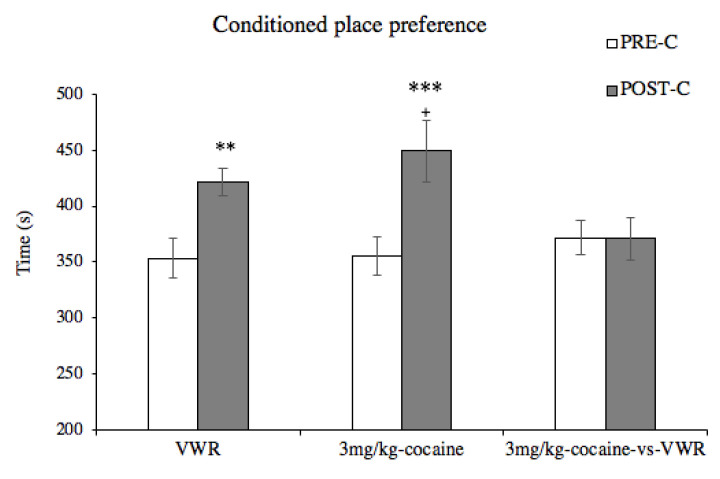
Voluntary wheel running induced CPP and neutralized the development of a preference for an effective dose of cocaine (3 mg/kg). The bars represent the time (s) spent in the active-wheel-paired compartment (VWR group) or in the cocaine-drug-paired compartment (3 mg/kg-cocaine and 3 mg/kg-cocaine-versus-VWR groups). White bars represent the time (s) spent during the Pre-C test and dark gray bars, after conditioning sessions in the Post-C test; ** *p* < 0.01; *** *p* <0.001 significant difference in the time spent in the paired compartment with respect Pre-C; + *p* < 0.05 differences in the time spent during the Post-C between the groups of 3 mg/kg cocaine and 3 mg/kg cocaine vs. VWR.

The ANOVA performed for these CPP data (see Figure 10) showed a significant effect of the factor days [F(1,50) = 13.533, *p* < 0.001] and the interaction days × CPPModality [F(2,50) = 3.571, *p* = 0.036]. The post hoc analysis showed that animals conditioned with a voluntary running wheel associated with one compartment and a blocked (inactive) wheel in the other compartment spent a significantly longer time in the active wheel compartment in the Post-C than in the Pre-C (*p* < 0.01). Similarly, animals that underwent a CPP protocol induced by 3 mg/kg of cocaine, which were controlled with an injection of saline in the non-drug paired compartment, developed CPP for the drug-paired compartment (*p* < 0.001). However, when one compartment was associated with a running wheel and the other with 3 mg/kg cocaine, no significant changes in the time of permanence of each compartment were observed. Additionally, our analysis showed that the time spent in the drug-paired compartment for animals conditioned with 3 mg/kg of cocaine in one compartment and saline in the other was significantly higher when compared to the time spent in the drug-paired compartment by animals also conditioned with 3 mg/kg in one compartment and a running wheel in the other. 

## 4. Discussion

The first aim of this study was to evaluate the effect of physical exercise on social-stress-induced increase of the conditioned rewarding effects of cocaine mediated by the increased neuroinflammatory response. The results showed that VWR increased cocaine-induced CPP regardless of the social-defeat-stress exposure. For the animals housed in the standard condition that are considered sedentary, only the ones that experienced intermittent social-defeat stress developed CPP for a non-effective dose of cocaine. However, the increase of physical activity by housing mice with one (1 × 4) or four (4 × 4) running wheels in their home cage induced the development of conditioned preference for the cocaine-paired compartment in non-stressed, as well as in defeated, mice. Only when the access to VWR was limited and intermittent was the exercise capable of blocking the increase in cocaine-induced CPP. When effective doses of cocaine were used, intermittent VWR modulated the memories associated with the drug. While VWR increased the time needed to extinguish the preference in non-stressed animals, it also reduced the time needed to achieve extinction in defeated mice. With respect the neuroinflammatory response, all the modalities of WVR increased the expression of IL-6 levels in plasma and also in the striatum and hippocampus. However, the results regarding the second aim of the study showed that VWR was, in general, an effective intervention to reduce hormonal response to social defeat, although corticosterone levels were higher in those animals that compete for the use of the exercise wheel (1 × 4 group). As a third aim, we showed the potential of physical exercise to increase the expression of BDNF, as access to VWR induced a very intense increase in BDNF levels, irrelevant of the stress experience, in both the striatum and hippocampus. As final aim, we showed that VWR can act as a hedonic substitute that competes with the rewarding potential of cocaine in the establishment of conditioned drug memories.

### 4.1. Only Intermittent VWR Counteracted the Increased Cocaine CPP Induced by Social Defeat 

Animals housed in sedentary condition presented a significant increase of plasmatic corticosterone levels measured 30 min after the last agonistic encounter when compared with the hormone levels of animals that had undergone a similar experimental manipulation but without experiencing social defeat. This rise in corticosterone levels was not observed in socially defeated animals housed with four activity wheels (4 × 4-RSD) or with intermittent access to VWR (INT–RSD). These results are in agreement with those of previous studies that also showed that regular physical exercise blunts the neuroendocrine response to stressors and promotes a decrease in corticosterone response to acute-stress challenges [10,58]. Physical exercise acts as an acute stressor [10,59], but after repeated exposures, it promotes an adaptation of the hypothalamic–pituitary–adrenal axis, thereby decreasing reactivity to further challenges [58,60,61]. Therefore, free access to physical activity counteracts corticosterone increase after social defeat. However, we did not record this protective effect in the groups of animals housed with one activity wheel (1 × 4-RSD). We hypothesized that, as animals had only one activity wheel per cage with four mice, there was a competition for that resource that also causes stress. In fact, other researchers have previously described that limited environmental-enrichment items can promote aggressive competition [62,63]. Additionally, this aggressiveness may be highlighted by the fact that animals being denied access to the resource can also display increased aggression as an indicator of exercise withdrawal [64].

Despite that the majority of VWR modalities analyzed promoted a decrease in the endocrine response to social stressors, we did not observe a protective effect over the enhancement of drug reward induced by RSD. We found that all the animals with continuous access to running wheels developed CPP for a subthreshold dose of cocaine. In light of previous reports, we only expected to observe CPP in socially defeated mice, as intermittent social stress has been proven to sensitize the reward system by increasing cocaine-conditioned rewarding effects [33,34,37,38]. However, among non-stressed groups, those exposed to continuous VWR showed higher CPP than those in the sedentary condition or with intermittent access. Not only did continued VWR schedules not protect against the increase in cocaine reward induced by social defeat, but it somehow enhanced the development of the place-preference conditioning. In fact, there are several studies that also found an enhancement of the formation of morphine- and cocaine-contextual-associated memories after VWR [14,25,26]. However, it is necessary to emphasize that there are also many studies that have reported a protective effect with decreased CPP of cocaine, ethanol, or morphine on animals with access to running wheels [21,22,23,24]. More consistent results are reported in studies that employ a self-administration paradigm. A majority of these papers point out that physical activity reduces the consumption of drugs such as methamphetamine, cocaine, and heroin [14,15,16,17,18] and prevents the reinstatement of drug-seeking behavior after extinction [16,19]. In fact, we recently published a study in which we found that intermittent VWR was capable of blocking the increase in ethanol consumption induced by social defeat on a self-administration paradigm [11]. We suggest that, even if both paradigms evaluate associative drug learning, CPP evaluates respondent conditioning, and self-administration is based on instrumental conditioning, which involves distinct mechanisms and brain regions [65]. Another reason that probably explains these discrepant results could be the different access pattern to VWR. Intermittent and controlled access to the wheels showed a protective effect to stress-induced increase in cocaine preference. Those defeated mice exposed to intermittent VWR did not develop a preference for a non-effective dose of cocaine. However, a non-significant increase in the time spent in the cocaine-paired compartment during the Post-C test was observed in the non-stress group with intermittent access, pointing to the ability of physical exercise to increase contextual associative memories. 

### 4.2. VWR Increased BDNF and Neuroinflammatory Response

Several mechanisms could underlie the enhancement exerted by physical activity on the potential of cocaine to induce CPP. Physical activity in the three different configurations analyzed (1 × 4, 4 × 4, and INT) promoted an elevation of striatal and hippocampal levels of the pro-inflammatory cytokine IL-6, an inflammatory effect that was exacerbated in animals that also experienced intermittent social-defeat stress. We previously reported that increases of IL-6 can sustain the enhanced response to cocaine in CPP [37]. There is a vast body of scientific publications that confirm the inflammatory effects of social-defeat stress and its potential to induce a sensitization of the immune system to further inflammatory challenges [37,66,67]. Moreover, the susceptibility of an individual to display inflammatory states after stress experiences has been directly related to the susceptibility to develop stress-associated pathologies (see revision in Reference [48]). Given its promising effects in blocking the endocrine response to stressors, we expected that VWR would also have a buffering effect against the sensitization of the immune system induced by social stress; however, our results point in the opposite direction. While some studies and revisions have highlighted the anti-inflammatory effects of exercise in both humans and rodents [68,69], these beneficial effects seem to be dependent on the exercise intensity, duration, and initial inflammatory state [70,71,72,73]. 

During physical effort, there is a peak of release of IL-6 by muscles that rapidly decreases after exercise [73,74,75,76], and its function has been connected with muscle metabolism processes [77]. This acute pro-inflammatory signaling from exercise is followed by a transient anti-inflammatory state caused by the induction of anti-inflammatory cytokines such as IL-10 and by the inhibition of the synthesis of other pro-inflammatory cytokines such as TNF-α [78,79]. This anti-inflammatory post-effect of exercise has been proven to be effective to regulate inflammatory patterns in states with elevated chronic inflammation, such as in depression, or in type 2 diabetes or obesity [70,79,80,81,82,83]. In subjects with homeostatic levels of inflammatory markers, it seems that the volume of exercise and the intensity determines whether it exerts an anti-inflammatory or an inflammatory effect [84]. Increases in intensity and volume are translated into increases in IL-6 plasmatic concentrations, which can modify the net result of this response, turning an initial anti-inflammatory effect into an inflammatory effect [85]. In an interesting study with humans, Paolucci and collaborators [86] found that, while moderate exercise reduced TNF-α and improved mood, high-intensity exercise promoted higher levels of perceived stress and inflammation. 

Some researchers claim that wheel-running behavior in mice shares some characteristics of a stereotypical or addictive behavior, which also can turn into maladaptive and obsessive behavior [87]. For instance, in a regimen of limited hours of access to food, animals will choose to run on the wheel rather than spend the time eating [88]. Therefore, it is likely that the enhancement of central and peripheral IL-6 levels registered in animals with access to voluntary running wheels is caused by a pattern of excessive exercise. In a previous research study, we found that increased IL-6 signaling can predict stress susceptibility to develop a sensitization to the rewarding effects of cocaine, while the prevention of this inflammatory state reversed the stress enhancement of cocaine CPP [37]. Thus, the enhanced inflammatory state induced by excessive VWR could help to explain CPP development. Unfortunately, the running wheels employed in the present study do not allow us to determine the intensity of the exercise performed by each mouse; this is a limitation to consider in the present study.

Another possible factor that could explain this increased CPP is the enhanced BDNF levels in the hippocampus and the striatum of animals under physical activity. Physical activity in running wheels has been previously liked to increase levels of BDNF [14,89,90], and accordingly, we found increases of BDNF in the striatum and the hippocampus of animals exposed to VWR, with even higher hippocampal levels in animals housed with one activity wheel (1 × 4). BDNF sustains the plastic process in the brain, as well as neurogenesis [91], and enhanced levels of this neurotrophin, mainly in the hippocampus, have been linked to enhanced cognitive performance with enhanced memory consolidation and learning [92]. As CPP establishment is sustained by an associative learning process between the interoceptive response and environmental cues [50], an enhanced capacity of learning and memory would impact CPP outcomes [14]. In fact, some researchers have previously reported that experiences of voluntary exercise increased BDNF in the hippocampus and promoted grater magnitudes of CPP induced by morphine [25] and cocaine [14]. While a deep discussion is out of the scope of the present study, is it worth mentioning that there is complex modulatory bidirectional mechanism between cytokines and BDNF [93]. Exercise stimulates the production of myokines, such as irisin, that have been related to an increase of BDNF levels. It also promotes the increase of inflammatory markers, such as IL-6, that can suppress BDNF expression [94,95], and in turn, BDNF levels also participate in the modulation of the neuroimmune axis [95].

### 4.3. VWR Favor Extinction of Conditioned Cocaine Memories Only in Defeated Mice

Although these results suggest that VWR increases sensitivity to the conditioned rewarding effects of cocaine, this should not mean that physical exercise increases susceptibility to cocaine addiction. In fact, physical exercise can also promote positive outcomes, such as increased learning, which may also accelerate the extinction of context-associated drug memories, stimulating the creation of new memories of the non-availability of drug reward. This perspective was explored in our second study.

As expected, 10 mg/kg was an effective cocaine dose that induced CPP in all experimental groups. When analyzing the time required to extinguish this preference, we the found that those animals with a previous history of social defeat (RSD–SED.Post) required more extinction sessions than those in the control stress group (EXP–SED.Post). This result was not surprising, as, in previous published works, we had already reported and discussed the potential of social defeat to increase the duration of drug-associated memories [37,38,96,97].

Taking into consideration the increase in BDNF levels observed in Study 1, we expected that an increase of physical exercise during extinction would potentiate associative learning, and therefore, a faster extinction of drug-associated memories. In fact, the majority of papers point that the moment when wheel running can exert its maximum benefit is after the acquisition of drug memories. For instance, Solinas and coworkers [98] showed that, after cocaine CPP establishment, animals housed in an enriched environment with free access to activity wheels were protected against cocaine-primed reinstatement of the conditioned preference. Similarly, Sikora and coworkers [99] found that animals housed with access to activity wheels after methamphetamine, nicotine and heroine acquisition of self-administration behavior reduced drug-seeking behavior during abstinence.

However, we found different effects depending on the stress background of the experimental subjects. While we observed a faster extinction in socially defeated animals with a regimen of intermittent VWR (RSD–INT.Post) when compared to their sedentary counterparts (RSD–SED.Post), we observed the opposite effect in non-stressed animals. Non-stressed animals with intermittent VWR (EXP–INT.Post) required more extinction sessions to abolish the conditioned preference than non-stressed sedentary animals (EXP–SED.Post). In comparable studies carried out with male mice and a similar CPP protocol with a dose of 10 mg/kg cocaine Mustroph and collaborators [23,26] found that the access to VWR after CPP protocol exerted a robust effect of reducing drug-associated memories. When discussing their results Mustroph and collaborators [23,26] also pointed that this decrease of CPP was linked to VWR pro-cognitive effects, which were linked to a BDNF upregulation. The main difference between their experimental design and ours is the inclusion of the variable stress, which has also been demonstrated to modulate BDNF expression in the mesolimbic circuit [100], and this factor may be underlying observed discrepancies between the two different stress conditions and between previous published studies.

### 4.4. VWR Acts as an Alternative Reinforcer for Cocaine-Induced CPP

In the third study, we explored the possibility that VWR not only affects learning processes, but also modulates the perception of intrinsic reward of cocaine. Our results confirmed that VWR has a rewarding effect that is able to counteract the cocaine potential to establish CPP. First, we confirmed, following the same conditioning procedure used for cocaine, that VWR has enough rewarding potential to promote place preference for the wheel-paired compartment in naïve animals without previous experiences with activity wheels. This result confirmed previous results of other researchers showing that rats and mice learn to lever-press for access to a running wheel [101] and develop CPP to the after-effects of running episodes [102,103]. The interesting result of our study is that these rewarding properties are strong enough to compete against the conditioned rewarding properties of an effective dose of cocaine (3 mg/kg). When this dose was associated with one compartment and controlled with an injection of saline in the other, cocaine induced a strong preference for the drug-paired compartment, a phenomenon that has also been reported in previous studies [39]. However, when one compartment was associated with 3 mg/kg of cocaine and the other with a VWR experience, animals did not develop a conditioned preference for any compartment. As we previously discussed, physical exercise is considered an alternative reinforcer, but the net effect of physical-exercise intervention, whether it can exert a protective effect acting as a hedonic substitute or enhance drug reward, seems to be related with the moment in which the intervention is applied. 

Our results endorse that VWR activates the same brain circuits as other natural rewards and drugs of abuse [104,105]. Access to reinforcers cause neuroplasticity in the mesolimbic dopaminergic system, which promotes the maintenance of rewarding behaviors [106]. Akin to our results, Lynch and coworkers [10] found that VWR, rather than having a protective effect, increased ethanol preference. They posited that this effect was caused by the fact that, if physical exercise is experienced before contact with the drug, it sensitizes and prepares the reward system to the rewarding effects of ethanol, and these effects act as a hedonic substitute of wheel running. However, when the first experience with VWR is concurrent with the drug, it exerts a protective effect, as it competes with drug potential to establish context memories, as we found in Study 3. Other studies have also reported protective effects of concurrent access to wheel running during drug-memory acquisition, such as the suppression of cocaine self-administration [107] and decreased cocaine-seeking behavior during extinction [108].

## 5. Conclusions

To sum up, our results showed that an increase of regular physical exercise through VWR experiences blunted the secretion of corticosterone in response to RSD stress. However, in spite of this decrease in the endocrine response to social stress, animals exposed to continuous physical activity were not protected against the stress-induced enhancement of the conditioned rewarding effects of cocaine. In fact, only intermittent access to VWR decreased mice’s sensitivity to the conditioned rewarding effects of cocaine. This result is relevant because contextual cues associated with the drug play a critical role in the development and maintenance of addictive disorders and can also promote relapse. This increase in vulnerability to develop cocaine-induced CPP can be promoted by the inflammatory potential of physical exercise and its improvement over associative learning through BDNF actions in the hippocampus and the striatum. Additionally, this effect may also be caused by a direct sensitization of the reward pathway by VWR, which, in Study 3, proved to be a powerful natural reinforcer. When applied after the establishment of drug-to-context associations, VWR exerted a dual effect depending on the stress background of the animal, as it decreased the number of extinction sessions in stressed animals, whereas it was increased in non-stressed mice. In this regard, further exploration of the interaction between social-stress experiences and the role of physical exercise in drug-memories extinction should be carried out in order to create a better understanding and to ensure the optimal exercise benefit. 

Together, these results highlight that physical exercise deeply impacts the organism’s response to stress and drugs. Thus, we believe that a good design of a physical intervention should take into consideration factors such as the duration of the exercise in order to be an effective tool in the management of stress and abstinence. This research work would help to optimize the designs of physical interventions, as it increases our understanding of the neurobiological effects of physical exercise.
